# Using Fitbit as an mHealth Intervention Tool to Promote Physical Activity: Potential Challenges and Solutions

**DOI:** 10.2196/25289

**Published:** 2021-03-01

**Authors:** Guilherme M Balbim, Isabela G Marques, David X Marquez, Darshilmukesh Patel, Lisa K Sharp, Spyros Kitsiou, Sharmilee M Nyenhuis

**Affiliations:** 1 Department of Kinesiology and Nutrition College of Applied Health Sciences University of Illinois at Chicago Chicago, IL United States; 2 Rheumatology Division, School of Medicine, University of Sao Paulo Sao Paulo Brazil; 3 Department of Medicine College of Medicine University of Illinois at Chicago Chicago, IL United States; 4 College of Pharmacy University of Illinois at Chicago Chicago, IL United States; 5 Department of Biomedical and Health Information Sciences College of Applied Health Sciences University of Illinois at Chicago Chicago, IL United States; 6 Division of Pulmonary, Critical Care, Sleep and Allergy Department of Medicine University of Illinois at Chicago Chicago, IL United States

**Keywords:** physical activity, fitness trackers, Fitbit, smartphones, interventional studies, adults, older adults, wearable, intervention

## Abstract

Consumer-based physical activity (PA) trackers, also known as wearables, are increasingly being used in research studies as intervention or measurement tools. One of the most popular and widely used brands of PA trackers is Fitbit. Since the release of the first Fitbit in 2009, hundreds of experimental studies have used Fitbit devices to facilitate PA self-monitoring and behavior change via goal setting and feedback tools. Fitbit’s ability to capture large volumes of PA and physiological data in real time creates enormous opportunities for researchers. At the same time, however, it introduces a number of challenges (eg, technological, operational, logistical), most of which are not sufficiently described in study publications. Currently, there are no technical reports, guidelines, nor other types of publications discussing some of these challenges and offering guidance to researchers on how to best incorporate Fitbit devices in their study design and intervention to achieve their research goals. As a result, researchers are often left alone to discover and address some of these issues during the study through “trial and error.” This paper aims to address this gap. Drawing on our cumulative experience of conducting multiple studies with various Fitbit PA trackers over the years, we present and discuss various key challenges associated with the use of Fitbit PA trackers in research studies. Difficulties with the use of Fitbit PA trackers are encountered throughout the entire research process. Challenges and solutions are categorized in 4 main categories: study preparation, intervention delivery, data collection and analysis, and study closeout. Subsequently, we describe a number of empirically tested strategies used in 4 of our interventional studies involving participants from a broad range of demographic characteristics, racial/ethnic backgrounds, and literacy levels. Researchers should be prepared to address challenges and issues in a timely fashion to ensure that the Fitbit effectively assists participants and researchers in achieving research and outcome goals.

## Background

The 2018 Physical Activity Guidelines for Americans Scientific Advisory Committee report shows strong evidence of the role of physical activity (PA) in reducing the risk of chronic diseases [1]. PA guidelines recommend that adults should engage in 150 to 300 minutes of moderate-intensity, or 75 to 150 minutes of vigorous-intensity, aerobic PA each week. However, only 53.3% of adults in the United States meet this guideline [1,2], while globally, 27.5% of adults are insufficiently active [3].

In 2019, fitness trackers and smartwatches (eg, Fitbit, Apple watch, Garmin), hereby termed PA trackers, were the number one fitness trend, with 19% of Americans owning at least one [4]. PA trackers measure a variety of variables including daily steps, intensity-specific minutes of PA, heart rate, calorie expenditure, stairs climbed, sedentary behavior, sleep duration, and sleep performance [5,6]. These devices are worn at various body locations, with hip, wrist, and thigh being the most common placements [6]. These commercially available PA trackers have been used in research studies to promote and monitor PA and in clinical practice for patients with chronic illness(es) where PA has shown to have a positive impact [7].

Among several brands of commercial PA trackers, Fitbit has emerged as one of the most popular in the wearable industry, having sold more than 100 million devices worldwide [8]. According to a recent International Data Corporation report on wearable devices, Fitbit is one of the leading companies in the wearable PA tracker space and has a large user base with more than 28 million active users [9]. A similar dominant trend is also observed in research, where Fitbits are the most frequently used devices, particularly in interventional studies that focus on promoting PA and other healthy lifestyle behaviors [10]. A search on the Fitabase library [11], which maintains a list of published research studies that have used Fitbit PA trackers, yielded over 200 validity studies and 172 interventional studies (this list is not exhaustive). Despite the large number of studies that have used Fitbit devices for research purposes, no prior publication has offered detailed insights regarding some of the challenges and limitations associated with their use in research studies for data collection, behavior change, and outcome assessment. Furthermore, there are no published technical reports or best practice guidelines to support researchers on how to best incorporate these devices in their study design and intervention. As a result, researchers are often left alone to discover and address some of these issues through “trial and error.” This paper aims to address this important gap by describing key challenges and solutions associated with the use of Fitbit PA trackers. While we recognize that this paper might be applicable to other commercially available PA trackers, a direct comparison with them or attempt to explain how these challenges and solutions may apply to other PA trackers goes beyond the scope of this paper.

Over the years, our research team has led several research studies utilizing Fitbits as intervention tools. Based on our collective experience with these studies, we identified several challenges that researchers need to take into account when designing and conducting studies that utilize Fitbit PA trackers. These challenges arise in distinct stages of a study, including study preparation, intervention delivery, and data management, and may have a significant impact on the conduct of the study and analysis of results. With the experience acquired, we describe empirically tested strategies and solutions that were effective in our interventions that included a broad range of demographic characteristics, racial/ethnic backgrounds, and educational levels.

## Description of the Studies

Between our team, we have conducted 4 separate studies utilizing Fitbit PA trackers. In this paper, we reference our experience from these studies to describe the key challenges associated with the use of Fitbits in research and subsequently summarize the strategies and remedies we used in each of these studies to address each challenge. Briefly, the included studies are (1) BAILA TECH [12,13], a Latin dance program for middle-aged and older Latinos; (2) ACTION [14], a lifestyle PA program for African American women with asthma; (3) Virtual Coach Study [15], a text-based, goal-setting intervention for adults with chronic diseases; and (4) iCardia4HF [16], a patient-centered, mobile health (mHealth) technology intervention to promote self-care in adult patients with chronic heart failure (HF). Table 1 depicts the main characteristics of each study.

**Table 1:** Characteristics of the studies.

Characteristics			Study						
			BAILA TECH [12,13]		ACTION [14]		Virtual Coach [15]		iCardia4HF [16]
Study Design			Single-group pre-post feasibility		Randomized controlled trial		Single-group pre-post exploratory		Randomized controlled trial
**Participants**									
	Sample size	20		53 (N_intervention_=25, N_control_=28)		30		25 (N_intervention_=11, N_control_=14)	
	Age (years), mean (SD)	67.0 (7.1)		43.4 (12.2)		47.0 (9.5)		56.0 (8.3)	
	Gender (female), n (%)	15 (75)		53 (100)		21 (75)		11 (44)	
	Race/ethnicity	Latinos		African American		African American (79%) and Latinos (18%)		African American (92%)	
	PA^a^ status	Low-active		Low-active		Sedentary		Sedentary	
	Health status	Healthy		Uncontrolled asthma (Asthma Control Test <20)		Chronic diseases (eg, hypertension, asthma, type 2 diabetes)		Chronic heart failure	
**Intervention**									
	Type	BAILAMOS dance program [17]		Modified version of the Women’s Lifestyle Physical Activity Program [18]		Remote goal setting and self-monitoring		Patient-centered mHealth^b^ technology intervention to promote adherence to self-care in patients with chronic heart failure	
	mHealth components	Fitbit Charge 2, Fitbit mobile app, text messages		Fitbit Charge HR, Fitbit mobile app, text message		Fitbit tracker (participants were given a choice of the following trackers: Alta, Charge 2, or Charge 3), Fitbit mobile app, text messages		Fitbit Charge 2, Fitbit mobile app, Withings Body Cardio scale, Withings Blood Pressure cuff, Health Mate mobile app, Heart Failure Health Storylines mobile app, text messages	
	Length (weeks)	16		24		8		8	
	Frequency	2x/week		5 monthly group sessions (barriers for exercising with asthma, Fitbit usage, and review		N/A^c^		Daily	
	Duration	30 min Fitbit instructional session + 60 min dance + 30 min technological troubleshooting (optional)		120 min		N/A		N/A	
Comparator			N/A		Fitbit Charge HR + PA educational material		N/A		Usual care (outpatient follow-up at the Heart Failure Center 7 days after hospital discharge and every 3 months thereafter)
Outcomes			Feasibility and acceptability of the BAILA TECH intervention, PA levels (eg, steps/day, engagement in moderate-to-vigorous PA/week)		Feasibility and acceptability of the modified Women’s Lifestyle Physical Activity Program, PA levels (eg, steps/day, low PA, moderate-to-vigorous PA/week, sedentary time), Asthma Control questionnaire [19], Mini-Asthma Quality of Life [19]		Feasibility and acceptability of the Virtual Coach intervention, average steps per week		Feasibility and acceptability of the intervention, heart failure self-care [20], PA levels (steps/day and moderate-to-vigorous PA/week)

^a^PA: physical activity.

^b^mHealth: mobile health.

^c^N/A: not applicable.

The BAILA TECH study [12,13] delivered the BAILAMOS Latin dance program tailored to middle-aged and older Spanish-speaking Latinos (for details, see Marquez et al [17]) infused with mHealth components [12,13]. The BAILAMOS dance program had 60-minute classes twice a week for 16 weeks and consisted of 4 Latin dance styles (Merengue, Bachata, Cha Cha Cha, and Salsa). For BAILA TECH [12,13], before each dance class, participants also attended 30-minute Fitbit instructional sessions. After each dance class, the research team was available for 30 minutes in case participants needed more assistance for troubleshooting technological issues. Participants were asked to wear their Fitbit for 2 weeks prior to intervention start, throughout the intervention period (16 weeks), and 1 week after the intervention, totaling 19 weeks.

The ACTION study delivered a modified version of the Women’s Lifestyle Physical Activity Program [14]. The original program was a 24-week, evidence-based, moderate-intensity walking intervention designed for urban mid-life African American women including (1) tailored walking prescription, (2) PA self-monitoring tool (pedometer), (3) group discussions to address goals and barriers, and (4) motivational telephone calls. The program was adapted to account for the asthma barriers to PA. The modified version was a 24-week intervention and included (1) a Fitbit Charge HR device, (2) one 2-hour educational session at the start of the intervention dedicating approximately 45 minutes to asthma education (based on the American Lung Associations Asthma Basics course) and approximately 30 minutes to Fitbit orientation, (3) weekly motivational text messages (average of 3 per week), and (4) monthly 2-hour group sessions to address barriers of exercising with asthma, answer Fitbit-related questions, review personalized step goals, and walk as a group. Participants were asked to wear their Fitbit throughout the intervention period (24 weeks).

The Virtual Coach study [15] delivered remote goal setting based on text messages and self-monitoring. Each participant was assigned to a coach and set individual daily goals with their coaches. Weekly goals outlined specific days, times, and number of steps for each of the 8 weeks of intervention. Participants were asked to evaluate how confident they were on reaching each goal (scale of 0-10). If a participant rated the confidence less than 8, coaches texted with the participant to modify the goal until confidence ratings were between 8 and 10 (highest confidence). Participants were asked to wear their Fitbit throughout the intervention period (8 weeks).

The iCardia4HF study [16] delivered a patient-centered, mHealth technology intervention to promote adherence to self-care in patients with chronic HF. The intervention lasted for 8 weeks and consisted of (1) the Heart Failure Health Storylines mobile app, (2) 3 connected health devices (Fitbit Charge 2, Withings Body Cardio scale, and blood pressure monitor) that connect to the app to facilitate self-monitoring of multiple HF-related parameters (eg, heart rate, weight, blood pressure, and PA), and (3) individually tailored text messages targeting health beliefs, self-efficacy, and HF knowledge. Participants were asked to wear the Fitbit for 8 weeks for 10 or more hours per day to monitor and gradually increase their daily steps.

## Challenges for Fitbit Use in Interventions and Potential Strategies and Solutions

### Overview

Challenges and solutions are classified into 4 main categories: study preparation, intervention delivery, data management, and study closeout. Two of these categories are subdivided according to the type of challenge presented. Within study preparation, we identified difficulties related to the Fitbit app and the user. During the intervention delivery, challenges were related to the interaction of participants with the Fitbit app and Fitbit tracker. We adopted this structure with the intention to provide a logical flow parallel to the conduct of a study (ie, study design, execution or management, and study closeout). Researchers should be aware that challenges related to intervention delivery and data management will occur concurrently during the study.

### Study Preparation

Selecting a PA tracker that aligns with study goals, participant preferences, and the data collection environment is essential and one of the first challenges a researcher might face early in the research process. Similar to other vendors, Fitbit has a large variety of trackers with different monitoring capabilities (eg, heart rate, sleep, automatic exercise tracking, geolocation), features (eg, automated notifications of sedentary behavior), and aesthetics (eg, large vs smaller screen and bands). All Fitbit models require that participants own a smartphone or other Bluetooth-enabled devices (eg, tablet) to synchronize data from the Fitbit tracker to the Fitbit app. Researchers should review the characteristics of available Fitbit models and, if possible, conduct formative research to gather data that align participants’ preferences with the study’s needs. For example, if exercise sessions or quality and duration of sleep are of direct interest to a study, then clip-on models (Fitbit Zip) that cannot automatically track these behaviors will not be a good fit for the purposes of the study. Successful strategies we adopted in our studies included focus groups [21], field usability testing followed by a pre-pilot study to assess the feasibility of using Fitbit trackers as part of the study intervention [22], and surveys [23] to evaluate participants’ preferences and perceptions about the use of Fitbit for PA self-monitoring. For example, before the main trial in 2 studies (ie, ACTION [14] and BAILA TECH [12,13]), we obtained input from focus groups regarding their familiarity with using PA trackers and if they would feel comfortable using these devices. In another study (ie, Virtual Coach Study [15]), we allowed the first few participants to try on different Fitbit models (Alta, Charge 2, and Charge 3) and solicited input from study participants to decide which model should be used in the preliminary study. In the iCardia4HF study [23], we conducted a cross-sectional survey to assess the perceptions and attitudes of patients with HF towards using various Fitbit trackers and mobile apps for self-monitoring of PA and other important measures (eg, heart rate).

After selection of the proper Fitbit model, researchers must decide how the Fitbit data will be collected from study participants during the study. Fitbit has a website dashboard in place that allows users to view and export cumulative data pertaining to their daily steps, intensity of activity, sedentary minutes, and other device-based measures. However, this dashboard is not suitable for research studies, particularly those with large sample sizes, because it does not allow concurrent access and exportation of data from multiple study participants. Data exportation is limited to the historic data of 1 participant at a time. Also, the Fitbit dashboard does not allow research staff to download intraday data such as minute-by-minute heart rate or steps, which can be used in a study as proxy measures to calculate Fitbit wear time (for more details, see the Data Management section) or to tailor the content of the intervention. For small studies (n≤20), manually checking and exporting data for 1 participant at a time may not be an important issue. However, for larger studies, this poses a significant barrier. Large studies require adequate strategies and robust mechanisms to remotely and automatically gather large amounts of incoming data from all participants in a single platform. Research teams have the option to utilize third-party commercial data collection platforms (eg, Fitabase) or research platforms (eg, iCardia [24], Mytapp) in order to remotely collect Fitbit data from participants’ accounts using Fitbit’s Web application programming interface (API). These platforms provide secure data acquisition and management tools that facilitate remote data collection from Fitbit PA trackers without the need for study participants to return the devices to researchers for data extraction. Another potential solution (if these platforms cannot support the needs of the study) is for researchers to develop their own data collection interfaces with the Fitbit cloud servers and data collection management environment for the purpose of their study using Fitbit’s API. However, this approach is time-consuming and requires sufficient programing expertise, including testing before utilization in the main study.

In our experience, the use of research platforms (eg, iCardia) that remotely collect Fitbit data from participants’ accounts proved to be an effective, reliable, and cost-effective method. The iCardia platform was successfully implemented in 3 of the 4 studies [9,10,25], allowing our research team to view and export Fitbit data from multiple participants and also to deliver personalized text messages based on the incoming data (for more details about the platform, please see Kitsiou et al [24]). iCardia is a secure, password-protected system that is hosted in a Health Insurance Portability and Accountability Act (HIPAA)-compliant server at the University of Illinois at Chicago. Currently, it is used in several studies funded by the National Institutes of Health [25-27]. iCardia provides researchers with a number of visualization and analytics tools (eg, graphs, charts, and dashboard reports) to facilitate monitoring of Fitbit data in real time (eg, steps, sleep, heart rate), thus alleviating the need for the tedious and costly return of devices to researchers.

Another research-based platform adopted by one of our studies (ie, Virtual Coach [15]) is the Mytapp texting platform. Mytapp is a low-cost, multilingual, adaptive text messaging application developed at the University of Illinois at Chicago for health behavior interventions. It includes numerous functions including individual and group messaging, scheduled messaging, recurrent messaging, surveys (with responses based on simple logic statements), message personalization, and data collection pertaining to number of daily steps (only) from Fitbit devices. Mytapp is currently utilized by numerous National Institutes of Health–funded studies and was utilized by the Virtual Coach study [15].

Fitabase is another well-known cloud-based platform that extracts and aggregates participants’ Fitbit data [28] and has been utilized in numerous intervention studies. Although Fitabase cannot send text messages to study participants via SMS, it has the ability to send notifications to Fitbit smartwatch devices (eg, Versa 2 and 3). None of our studies implemented Fitabase as a data collection environment. Nonetheless, a search with the descriptors “intervention AND Fitabase” on PubMed, Scopus, and ScienceDirect indicated 24 intervention studies using Fitabase since 2018.

Another challenge in the preparation phase is ensuring participants’ anonymity and privacy when creating Fitbit accounts. Fitbit accounts require a unique email address for each user. While collecting unprecedented amounts of data and conducting interventions with mHealth tools, researchers should be concerned with protecting the privacy of research participants [29]. Additional concerns arise because some mHealth technologies (eg, mobile apps) may transfer participants’ data to third-party companies for marketing purposes [29]. Therefore, if using third-party platforms, researchers are advised to check whether the platform fulfills all the necessary elements to ensure participants’ anonymity, privacy, and HIPAA compliance.

Our experiences directed us to a 2-step solution: (1) setting up anonymous commercial or institutional email accounts and (2) setting up deidentified Fitbit accounts for the purpose of the study. This individualized email address will then be linked to a Fitbit account in which personal identifiers should not be used. The same procedure should be adopted if using an authorized third-party data collection environment. Importantly, research staff and participants need to be advised that the email and Fitbit accounts should be used strictly for the study’s purpose. We suggest limiting the number of research staff responsible for linking personal identifiers with email and Fitbit account’s user information. Moreover, account information should be stored in protected computers within encrypted documents. This strategy was successfully implemented within our 4 studies.

Another critical step before intervention delivery is calibrating the Fitbit according to the participants’ characteristics (ie, height, weight, and stride length) and study needs (eg, exercise bout length, PA goals). This process should be conducted on an individual basis with the information inserted in the participant’s Fitbit account. Research staff who have access to participants’ personal identifiers can process the calibration at Fitbit’s website utilizing participants’ credentials (ie, anonymous Fitbit accounts). Importantly, participants should receive the Fitbit only after the calibration process is concluded. Specifically, ACTION’s study [14] participants had their stride length measured at baseline data collection. A research staff member entered the stride length information for each participant before distributing the Fitbits. If it is not possible to measure participants’ stride length, information on weight and height should be entered for each participant since this information acts as a proxy for stride length. In 1 of our studies (ie, BAILA TECH [12,13]), participants provided self-reported weight and height at baseline to calibrate their Fitbits.

Table 2 displays the challenges and solutions or strategies at the study preparation stage.

**Table 2:** Challenges and solutions or strategies for utilizing the Fitbit physical activity (PA) tracker in health interventions at the study preparation stage.

Challenges		Strategies and solutions
**Tracker and app related**		
	Selecting a PA tracker model that aligns with study goals and participant preferences	Select the proper model based on study goals and outcomes, using Fitbit’s online comparison tool [30]; conduct formative research (if possible) with potential participants to assess ease of use, comfort, and preferences.
	Selecting and setting up the data collection environment for the Fitbit data	Choose one of the following options: third-party or other existing research platforms (eg, Fitabase, iCardia), developing your own data collection platform for the purpose of your study, or using Fitbit’s platform and dashboard to export individual data.
	Setting up Fitbit study procedures	Develop a Fitbit manual of operations for research staff (setting up, pairing, and calibrating devices); devote sufficient time for training and also plan for frequent retraining of research staff on how to perform various Fitbit tasks and troubleshoot common issues (eg, Fitbit mobile app installation and setup, device pairing, and syncing), because of the numerous changes and updates that may occur during the study in the Fitbit app and devices; ensure anonymity of study participants by creating anonymous institutional or commercial email and Fitbit accounts.
	Setting up and pairing participants Fitbit: poor internet connection; participants’ smartphone and Fitbit compatibility issues (eg, outdated operating system, insufficient memory space, firmware updates)	Conduct the Fitbit set-up process with the assistance of trained research staff, test the quality of the internet connection at the location where the Fitbits will be set up, and check participants’ phone compatibility with Fitbit at enrollment to determine if updates are needed or if participant needs a study (loaner) phone.
	Fitbit calibration	Collect data on participants’ weight, height, and if possible, stride length and add the information to the Fitbit app before giving the Fitbit to the participant; personalize PA goals according to the study’s needs (eg, exercise bout length, goals) when possible.
**Participant related**		
	Participants’ unfamiliarity with Fitbit technology (unfamiliarity with PA tracker and app, concerns about privacy)	Develop a user manual according to participants’ reading level explaining how to use the device and mobile app in the context of the study; conduct Fitbit orientation sessions with study participants; consider a run-in period (eg, 1 week) to allow participants to familiarize themselves with the technology; identify a superuser (eg, family member or research assistant) to assist with and troubleshoot technology issues; assure participants that procedures to ensure anonymity and privacy are in place.

### Intervention Delivery

Before participants receive the Fitbits, the research staff need to be familiar with the Fitbit tracker and mobile app. To this end, staff members have to undergo sufficient training and experiential learning sessions on how Fitbit works and how to troubleshoot common issues (eg, Fitbit mobile app installation and setup, device pairing, and syncing). Given the frequent changes that may occur in the Fitbit app and devices during the study, researchers should also plan to have frequent retraining sessions. In addition to developing the training material, a manual of operations should be created and made available to all research staff that will interact with research participants. This strategy was successfully implemented with research staff in all 4 studies [12-16]. Issues with either the tracker or the mobile app will undoubtedly occur. Solving these issues as soon as possible is crucial to prevent data loss, which could potentially harm outcome assessment, fidelity, or feedback strategies that are dependent upon Fitbit data. Maintaining log sheets to record the date and characteristics of any technical issues occurring during the study and remedies applied to address them is strongly recommended.

Although technology use is familiar to many study participants, some segments of the population are not familiar with technological devices such as Fitbits and associated mobile apps. It is essential to be prepared for issues that may arise in relation to participants’ interaction with the Fitbit tracker and mobile app. Before launching the intervention, it is highly advisable to conduct an orientation session to teach participants how to use the Fitbit tracker and mobile app in the context of the study. Our experiences showed that conducting orientation sessions is essential for the success of the intervention. We carried out orientation sessions with middle-aged and older Latinos (ie, BAILA TECH [12,13]), African American women with asthma (ie, ACTION [14]), older adults with HF (ie, iCardia4HF [16]), and low-income, minority adults with chronic diseases (ie, Virtual Coach [15]). Importantly, we also noticed that educational classes during the intervention were essential to enhance participants’ engagement and satisfaction with Fitbit [31].

Additionally, the research team should distribute a user manual containing a guide on how to execute basic tasks required by the study, troubleshooting of common issues, and answers to frequently asked questions (Multimedia Appendix 1). The Fitbit User Manual available online [32] is a great resource to guide the development of a customized manual. Our studies utilized the Fitbit User Manual as a template. In BAILA TECH [12,13], ACTION [14], and iCardia 4HF [16], we added images and more in-depth information of Fitbit features that were essential to achieve the intervention goals and for the success of data collection (eg, syncing, charging). It is essential to use plain and accessible language according to participants’ educational attainment and literacy levels to facilitate comprehension of the manual. For example, in the BAILA TECH study [12,13], the manual was developed at a sixth-grade reading level.

Moreover, we recommend a run-in period (eg, 1 week) prior to baseline PA data collection to allow participants to familiarize themselves with the Fitbit device and its associated mobile app before the intervention starts. Run-in periods are useful in interventions with the use of technology since individuals often adopt and discontinue technology use at a different pace [33] and can flag the need for adaptations. If the study plans to collect PA data, the run-in period is essential. The first week is then disregarded, and the second week can be used for baseline PA assessment. This strategy was successfully adopted in the BAILA TECH study [12,13].

Identification of a superuser (eg, family member or research assistant) to assist with daily tasks and troubleshoot issues is also suggested. For example, the ACTION [14] and iCardia4HF [16] studies had 2 research assistants available to assist participants via text messages or phone, and if needed, in-person meetings were scheduled to troubleshoot. Nevertheless, researchers should be aware that providing assistance on an individual basis will likely increase participant-research staff interaction, which should be acknowledged as a potential confounder. In the ACTION [14] study, participants in the control group also received Fitbits; in such cases, the researcher should ensure that both groups receive equal technical support (ie, availability of research staff to troubleshoot) to avoid imbalances between the 2 groups.

The next step is intervention delivery, which presents several challenges. We classified these challenges in 2 categories: (1) participant-mobile app interaction and (2) participant-Fitbit tracker interaction. One of the most common issues we experienced in all 4 studies with participant-mobile app interaction was related to infrequent syncing of the Fitbit tracker with the Fitbit mobile app, which can lead to data loss if not identified and addressed in a timely manner. Fitbit trackers can hold up to 7 days of intraday data and up to 31 days of cumulative data in their memory without syncing. When a user syncs, the data are transferred through the phone to the cloud-based server, and the tracker’s memory is flushed to create space for the new data. If the user does not sync within 7 days, the intraday data of the first day since the last sync are erased to create space for the new data of the eighth day, and so on. Thus, the process of syncing becomes critically important in interventions using Fitbits, especially if Fitbit is used as both an intervention and outcome assessment tool.

Without syncing, the research team does not have access to participants’ data and runs the risk of data loss. Detecting participants who have not synced periodically (maximum of 7 days) and addressing the issue as soon as possible are very important and quite challenging in larger studies. In general, participants either forget to sync or forget how to go through the syncing process. The Fitbit app offers a “sync automatically” option as long as the Bluetooth and internet connections are enabled and the app is active or running in the background. However, we noticed that many participants tend to turn off their Bluetooth or the app to save battery on their phone and then forget to turn them back on or access the app to sync. To address this challenge, study participants should have easy access to information on how to sync the tracker with the mobile app (eg, manual). We also recommend that the research staff closely monitor participants’ data daily (eg, via the platform). It is easy to detect the participants who have not synced recently or if their Fitbit battery is low and subsequently send a notification (eg, via a text message, phone call, or email) to sync or charge their battery. Fitbit Charge 2 and other newer models display a battery notification on the tracker’s screen when the battery energy is below 20%. Therefore, a reminder from the research team may not be needed. In the BAILA TECH [12,13] and ACTION [14] studies, research assistants were tracking participants’ synced Fitbit data daily. After 3 days without syncing, research assistants contacted the participant via SMS reminding them to sync their Fitbit. The BAILA TECH study [12,13] delivered an intervention that allowed research assistants to meet participants twice a week, which made it easier to remind participants to sync their Fitbits in person. Another successful strategy we adopted in the iCardia4HF [16] study was to send weekly text messages to all participants reminding them to sync. A feature implemented in the iCardia platform allowed us to send an automated reminder to selected participants when their last sync time exceeds a prespecified number of days decided by the research team (eg, 6 days).

A less common issue that impacted our ability to collect Fitbit data from several participants for days, especially in the ACTION [14] and BAILA TECH [12,13] studies, was a firmware update. During this update programmed by Fitbit, several participants were not able to perform the update by themselves and were also not able to sync their trackers with the mobile app. One important issue observed in BAILA TECH [12,13] is that many participants did not have enough memory space in their phone to allow updates. We often reminded participants to delete files on the phone and make sure the updates were completed before it interfered with the app function. While the update did not change Fitbit features on the tracker and mobile app, it required the research teams to meet with participants to solve this issue. Although not common, researchers should be aware that such updates might occur in the middle of the study and disrupt data collection for some participants who are less adept with technology.

Frequent updates also occur on the Fitbit app, usually without prior notification to the research team. Some updates are minor, addressing software issues such as bugs. However, other updates, involving the addition or removal of behavior change features or tools, are more disruptive and may have significant implications for the design and execution of a study. For example, in the iCardia4HF study, changes to the app user interface while the study was ongoing required significant adjustments to study materials (eg, participants’ manual) and participant training or study onboarding sessions. Also, the addition of new features such as motivational messages (which now appear at the top of the dashboard every time a user accesses the Fitbit app), guided exercise programs, guided mindfulness and meditation, social media interactions, and user notifications may introduce new intervention components beyond those desired or anticipated by the research team, which may have an impact on the study design and results. It is important for researchers to understand and assess the impact that these changes may have in their study, in order to come up with a strategy to either deactivate such features when they become available (if possible to avoid contamination of their intervention) or interview participants at the end of the study to better understand which of these features were used, how often, and how they may have contributed to the outcomes of interest (eg, steps, moderate-to-vigorous PA, sedentary time, wear time, and engagement).

The interaction between participants and the Fitbit tracker presents, in general, challenges related to broken Fitbit trackers or charging cables and losing the Fitbit. Losing the Fitbit demands prompt actions from the research staff. A study that utilized Fitbit Zip to monitor PA levels during 16 weeks in 50 participants reported that 14 participants lost their Fitbits, and some participants (the exact number was not reported) had issues with charging cables [34]. The problem with broken or lost Fitbits is that, in some instances, participants do not report it to the research staff in a timely manner. Participants should receive instructions in the orientation session and in the manual about the importance of reporting a broken or lost Fitbit. As mentioned earlier, the research staff team should monitor participants’ incoming data frequently, which in the case of a broken or lost Fitbit, would flag that the participant is not syncing the Fitbit.

To account for lost and broken Fitbits, the researcher should ensure at least a 10% Fitbit and charger surplus in study budgets in case replacements are needed. For example, in the ACTION study [14], 6 Fitbits broke, and 8 chargers were broken or lost and had to be replaced. In the BAILA TECH study [12,13], 1 Fitbit and 1 charger had to be replaced. As we had a surplus, the replacements were made quickly. Researchers also should anticipate that some participants may not be satisfied with the band color or material and that some bands can become discolored. Dissatisfaction with the Fitbit color or material can lead to reduced wear-time adherence. Therefore, we also suggest that when acquiring the Fitbits, a 10% surplus of bands of different colors and materials (eg, hypoallergenic, metal) should be purchased. Studies with follow-up durations ≥12 months may require a higher surplus (eg, 20%) given the short life expectancy of these devices and bands.

Monitoring adherence to wearing the Fitbit tracker and identifying epochs of non-wear time are 2 of the most critical and challenging factors for studies that rely on Fitbit data to assess changes in PA outcomes and also to deliver a personalized behavioral change intervention. A key feature that is currently missing from all Fitbit trackers is an advanced wear-time sensor to automatically detect when the device is removed. Without knowing when and for how long a study participant wore the Fitbit, it is difficult to monitor compliance and determine with certainty whether, for example, decreases in the number of steps and moderate-to-vigorous PA are due to sedentary behavior or nonadherence to wearing the Fitbit. Unlike research-grade activity tracking devices (eg, ActiGraph GT3X+), Fitbit PA trackers do not offer a capacitive touch technology or specialized software that can help researchers analyze the raw accelerometer data and identify epochs of nonwear time.

To address this challenge, in the BAILA TECH [12,13], ACTION [14], and iCardia4HF [16] trials, we developed and tested a novel method. This method captures continuous heart rate sensor data measured at 1- to 5-second intervals and uses this data to calculate minutes of nonwear time based on the absence of a heart pulse for 60 or more seconds. Data collection and calculation are done by the iCardia platform using the Fitbit Web API. This method is susceptible to errors if a study participant is not properly wearing the tracker on the wrist (eg, too loose or too tight). Although, with adequate personnel training and monitoring, this method has allowed our research team to gather critical wear-time data to determine compliance and distinguish between real and spurious sedentary time [22]. Importantly, this method can only be implemented with Fitbit activity trackers that have a heart rate sensor (some Fitbit models do not have this feature).

Regardless of the type of issue faced during the intervention period, we learned a single strategy that helped to address most of the challenges during the intervention delivery: devoting time to in-person intervention meetings or placing telephone calls when necessary to discuss potential challenges or issues associated with the use of Fitbit. Across the studies we conducted, we dedicated a portion of the in-person meeting to Fitbit-related content [9,10,20,35]. During this time, participants were free to externalize any concerns or issues related to Fitbit use, and research staff were ready to assist with possible problems or questions. We also recommend that, at these meetings, research staff should be available to address issues on an individual basis since some might need extra time to be resolved.

We display a summary of the challenges and solutions or strategies at the intervention delivery stage of the study in Table 3.

**Table 3:** Challenges and solutions or strategies for utilizing the Fitbit physical activity (PA) tracker in health interventions at the intervention delivery stage.

Challenges		Strategies and solutions
**Interaction between participants and app**		
	Syncing-related issues include risk of data loss due to study participants forgetting to sync their Fitbit tracker with the mobile app, participants not knowing or remembering how to sync, Bluetooth turned off, internet turned off; login issues include logging off accidentally and forgetting credentials.	Offer written step-by-step instructions on how often and how to sync their tracker with the Fitbit mobile app to minimize data loss; send weekly text messages reminding all participants to sync their Fitbits or target only those who have not synced periodically^a^; email or text participants with step-by-step troubleshooting; contact the superuser; ask participants to write down login credentials in the manual or another place of easy access.
	Fitbit updates during the intervention	Notify participants about app updates; update the participants’ manual; determine how to handle the introduction of new features or behavior change techniques (eg, behavioral notifications about PA or CV^b^ health) according to study needs.
	Accidental deletion of Fitbit app	Contact the superuser (eg, family member or research assistant).
**Interaction between participants and tracker**		
	Broken Fitbit tracker (battery does not charge or broken or lost charger), lost Fitbit tracker	Ensure at least a 10% Fitbit and charger surplus to the study budget in case Fitbit needs to be replaced; have participants use a Bluetooth locator app to help find the device.
	Fitbit tracker aesthetics (width, color, discoloration of the band)	Offer multiple bands of different colors, sizes, and materials.
	Skin irritation and rashes caused by the Fitbit tracker	Purchase and offer hypoallergenic bands to participants that report skin irritation and rashes; suggest participants remove the tracker or wear the tracker on the other wrist for at least 1 hour per day.
	Adherence to Fitbit usage (Fitbit does not calculate wear time)	Choose a device with a heart rate monitoring feature to calculate an approximate wear time [24]; have research staff send messages if wear time <10 hours/day or build automated text message reminders to wear the Fitbit.

^a^Most Fitbit trackers can save up to 7 days of intraday data and up to 31 days of cumulative data in their memory without syncing. After that time, data are erased from the tracker to create space for the new data. This creates a risk for potential data loss if study participants do not sync their tracker regularly.

^b^CV: cardiovascular.

### Data Management

Although Fitbit provides a vast amount of data, some critical data (eg, wear time) are either not readily available through Fitbit’s Web API or not accurate. For those trackers that capture continuous heart rate, these data can be used as a proxy for wear time. Third-party authorized platforms (eg, iCardia) automatically calculate Fitbit wear time based on the incoming heart rate data. However, if using heart rate data directly from the Fitbit platform, research staff need to develop rules for determining wear time and manually identify gaps in heart rate that indicated nonwear time. This tedious and challenging process requires a considerable amount of time and is not advisable due to time requirements and increased likelihood of wrongly estimating wear time. Nonetheless, we recognize that this might be the only option due to Fitbit’s limitation in offering complete wear-time data.

The process of Fitbit data collection and management is continuous throughout the study and requires the use of several data exportation and organization practices by research staff members. Ideally, we recommend that data should be exported from the third-party platforms, researchers’ platform, or Fitbit’s website at least once a week. Data are exported in a comma-delimited format (.csv) and should be appropriately organized by research staff according to the study’s needs. In our experience with the BAILA TECH [12,13] and ACTION [14] studies, each week of data has a spreadsheet with all the variables of interest. This spreadsheet is comprised of daily data (ie, wear time; time spent in light, moderate, vigorous, and moderate-to-vigorous PA; and sedentary behavior) for each participant, as well as an average of each variable for each participant for that week and a grand average of all participants for that week. Importantly, the spreadsheet needs to determine valid days of data according to the wear-time rules previously adopted. Days deemed as nonvalid should not be included in participants’ average, following the standard practice of studies measuring PA with accelerometry [36]. For example, if in a given week, the participant had 5 valid days and 2 nonvalid days of data, the average should be calculated based on the 5 valid days. Once the intervention data collection with Fitbit is complete, a new master spreadsheet should be created with participants’ average of all the periods Fitbit was collecting data.

We display a summary of the challenges and solutions or strategies at the data management stage of the study in Table 4.

**Table 4:** Challenges and solutions or strategies for utilizing the Fitbit physical activity (PA) tracker in health interventions at the data management stage.

Challenges			Strategies and solutions
**Determining Fitbit wear time**			
	Individualized manual approach (not recommended for large studies)	Review participants’ daily heart rate data through the Fitbit platform to identify gaps indicating nonwear time and develop rules for determining adherence to wearing Fitbit.	
	Automated approach	Utilize an existing platform or application (eg, iCardia, Fitabase) that will automatically calculate Fitbit wear time based on the incoming heart rate data or develop your own application to remotely collect the data utilizing Fitbit’s Web API^a^.	
Accuracy of sedentary minutes calculated by Fitbit			Calculate adjusted sedentary time based on heart rate and wear-time data.
Data export			Export and organize participant data weekly and before study closeout.

^a^API: application programming interface.

### Study Closeout

Once the intervention is concluded, Fitbit data collection should cease. There are several actions that research staff should take to guarantee Fitbit data are not collected after the end of the study, which would violate institutional review board and HIPAA regulations. If the researcher is using a third-party platform, research staff should access each participants’ Fitbit account and revoke access to the third-party platform. Importantly, this action will not affect the ability to export data that were already shared with the platform, and it will only stop the sharing of incoming data. If participants are allowed to keep the Fitbit after the end of the study, it is necessary to replace the email associated with the Fitbit account with the participants’ personal emails. Research staff should provide orientation (eg, step-by-step document on how to replace the email) to study participants on how to dissociate the study’s email account with the Fitbit account, or the research team can do it for participants to ensure that the email is dissociated. For example, in the ACTION [14], Virtual Coach, and BAILA TECH [12,13] studies, we developed a guide on how to dissociate the study account email with the Fitbit account. Participants should be given a time period to replace the email (eg, 1 month). In case participants do not replace the email within the time period, the research team should delete the Fitbit account permanently.

We display a summary of the challenges and solutions or strategies at the study closeout stage of the study in Table 5.

**Table 5:** Challenges and solutions or strategies for utilizing the Fitbit physical activity (PA) tracker in health interventions at the study closeout stage.

Challenges	Strategies and solutions
Ensuring IRB^a^ compliance with data collection	If using a third-party platform, revoke access on Fitbit’s website at the end of the study, dissociate the study email on the Fitbit account, create step-by-step instructions on how to dissociate the study email account from the Fitbit account; if not dissociated, delete the Fitbit account; delete the study email account; follow IRB guidelines for data destruction.

^a^IRB: institutional review board.

## Discussion

In this paper, we described a number of key challenges associated with the use of Fitbit as an intervention or PA measurement tool. Difficulties with the use of Fitbit in research are encountered throughout the entire research process. Researchers should be prepared to address challenges and issues in a timely fashion to ensure that the Fitbit effectively assists participants and researchers in achieving the research goals. Most of the challenges can be addressed with proper staff training and a complete manual of operations.

We conceived and developed this paper to facilitate the understanding, uptake, and utilization of Fitbit as an mHealth tool to promote behavior change. While we aimed to present a series of challenges that researchers might encounter when using Fitbit in their research along with strategies to address these, we acknowledge that these strategies are not definitive. Their feasibility and effectiveness might vary depending on several factors (eg, funding, research staff experience and size, and research goals). Further, while our experience was with Fitbit PA trackers, many of the challenges and proposed solutions may be considered when using other commercial PA trackers, yet it was beyond the scope of this paper to make any direct comparison with them or attempt to explain how these challenges and solutions may apply to other PA trackers. We hope that our shared experiences will act as a guideline to help researchers to make conscious choices on the use of Fitbit in their research, as well as assist with more effective and intentional use of Fitbit in the research realm.

## Appendix

Multimedia Appendix 1 Fitbit manual from ACTION trial.

## Abbreviations

API: application programming interface
HF: heart failure
HIPAA: Health Insurance Portability and Accountability Act
mHealth: mobile health
PA: physical activity
