# Supplementary material for: Using Fitbit as an mHealth Intervention Tool to Promote Physical Activity: Potential Challenges and Solutions
Source: JMIR Mhealth Uhealth. 2021 Mar 1;9(3):e25289. doi: 10.2196/25289 (PMC7961407; doi:10.2196/25289)
Supplement: Multimedia Appendix 1 [file mhealth_v9i3e25289_app1.pdf]

FITBIT USE

What is a Fitbit?

A Fitbit is a tool that helps measure how many steps you take each day. It also can measure your heart rate, distance walked, calories burned, floors climbed and monitor the number of hours you sleep.

What will you receive?

You will be given a Fitibit Charge HR, a charge cable, a wireless sync dongle and a cleaning kit.

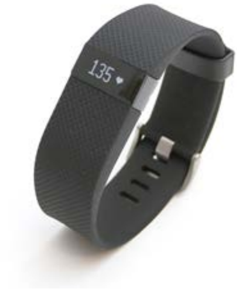

Figure 1. Fitbit Charge HR

Figure 2. Fitbit charge cable

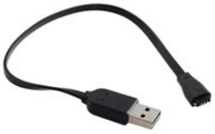

Figure 3. Wireless sync dongle

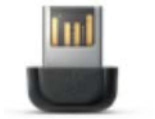

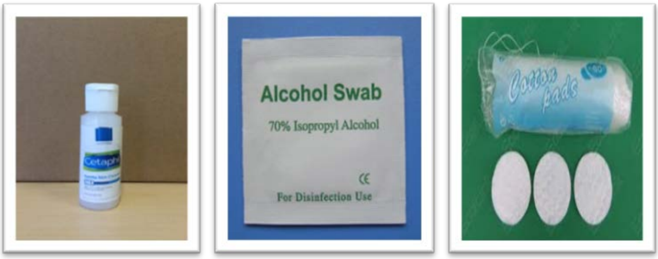

Figure 4. Cleaning kit

How to set up the Fitbit Charge HR account on your smartphone, computer or tablet

We have already set up your Fitbit account and downloaded the app onto your cell phone at your first study visit. Your Fitbit is already paired with the app and you are set to start wearing your Fitbit!

Just in case you need to log in again, here is your Fitbit account information.
Your Fitbit user name is:______________________

Password: ___________________

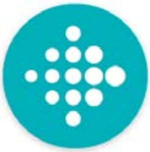

Figure 5. Fitbit app icon

When you open the Fitbit app on your phone you will see the dashboard. This means that the setup process is complete and you're ready to start moving. Your Fitbit will automatically start syncing.

Here what is your dashboard looks like:

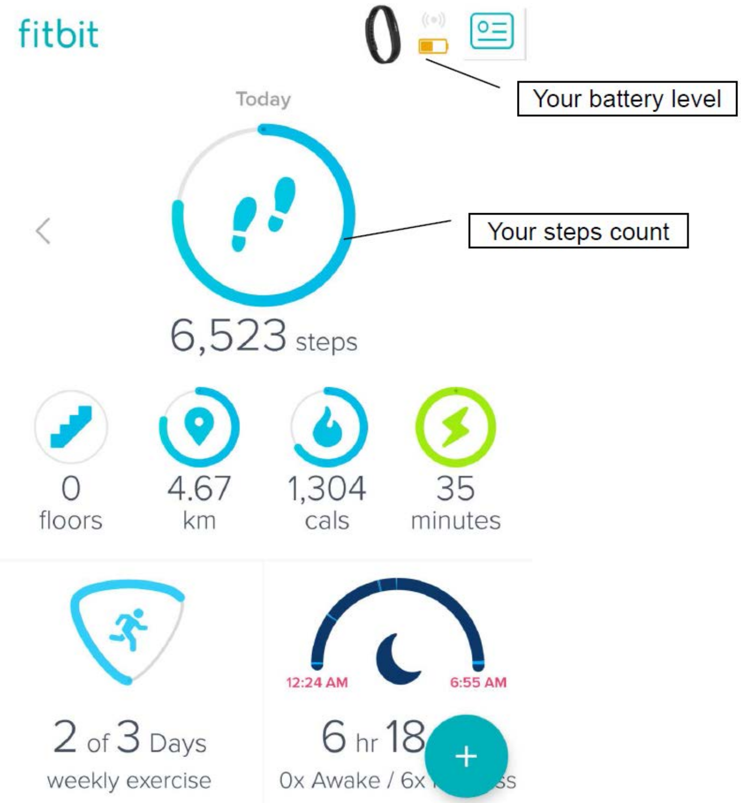

Figure 6. Fitbit app dashboard
